# A Preliminary Investigation of Myostatin Gene (*MSTN*) Variation in Red Deer (*Cervus elaphus*) and Its Implications for Venison Production in New Zealand

**DOI:** 10.3390/ani12131615

**Published:** 2022-06-23

**Authors:** Lily Cunningham, Huitong Zhou, Qian Fang, Mark Tapley, Jonathan G. H. Hickford

**Affiliations:** 1 Department of Agricultural Sciences, Lincoln University, Lincoln 7647, New Zealand; lilycunningham22@gmail.com (L.C.); huitong.zhou@lincoln.ac.nz (H.Z.); freeman.fang@lincoln.ac.nz (Q.F.); 2 Peel Forest Estate, Peel Forest, Geraldine 7992, New Zealand; mark@pfe.nz

**Keywords:** *Cervus elaphus*, *MSTN*, carcass, growth, New Zealand

## Abstract

**Simple Summary:**

Myostatin is a negative regulator of skeletal muscle growth. Despite variation in the myostatin gene having been reported in livestock species, little work has been undertaken in red deer (*Cervus elaphus*). This study describes the presence of two nucleotide sequences of the myostatin gene in New Zealand red deer, but no association was found between this variation and selected muscle and growth traits.

**Abstract:**

Myostatin (MSTN), also known as growth differentiation factor 8 (GDF-8), is a negative regulator of lean muscle tissue growth. Variation in the gene has been studied in many domesticated species, because of its potential to dramatically increase muscle mass. It has, however, not been investigated in red deer (*Cervus elaphus*). In this study, variation in *MSTN* intron 1 was investigated in 211 male New Zealand red deer, for which phenotypic measurements of *M. Longissimus dorsi* (eye muscle) (width, depth, and area, together with 12-month weight) were recorded. Two sequence variants (named *A* and *B*) differing by one nucleotide (c.373 + 224) were identified in the intron 1 region of the gene resulting in three genotypes (*AA*, *AB,* and *BB*; frequencies of 63.5%, 30.8%, and 5.7%, respectively), but no association between this variation and any of the quantitative measurements was detected. These results suggest that the deer *MSTN* is less variable than for other livestock species and that its activity may be controlled to maintain a size–growth equilibrium.

## 1. Introduction

Myostatin (MSTN), also known as growth differentiation factor 8 (GDF8), is a member of the transforming growth factor-β (TGF-β) family. Its main function is to act as a negative regulator of skeletal muscle growth and myostatin gene (*MSTN*)-deficient mice display an increase in skeletal muscle mass [1]. Accordingly, research on *MSTN* variation and its effect on meat production has been of great interest to livestock farming. Variation in *MSTN* has been associated with various meat production traits in cattle [2,3,4,5], sheep [6,7], goats [8,9,10], pigs [11,12], rabbits [13,14], chicken [15,16], and geese [17].

Red deer (*Cervus elaphus*) were first introduced to New Zealand in the 1800s (from England and Scotland) to provide hunting and game opportunities. Elk (*Cervus canadensis*) were also introduced from North America into Fiordland, and they underwent hybridization with the red deer in the wild to produce a hybrid known as Wapiti. Both are now considered pest species with vast numbers present throughout the country ([18]; https://www.doc.govt.nz/nature/pests-and-threats/animal-pests/deer/, accessed on 9 March 2022).

In order to control the wild populations in New Zealand, deer were at first shot by professional hunters, then the export of wild venison began in the 1960s. Industry pioneers saw an opportunity to build on this venison trade, and the live capture of wild deer started in the 1970s. These captured deer were farmed for venison and velvet production, creating a new livestock industry [19]. Since then, New Zealand has become the largest source of farm-raised venison globally ([19]; Deer Industry New Zealand (https://www.deernz.org/, accessed on 9 March 2022).

Venison is a red meat that is higher in iron and protein than other farmed red meats. The protein is more diverse in amino acid content and lower in total calories, cholesterol, and fat than most cuts of grain-fed beef, pork, or lamb (USDA Nutrient Database, NDB numbers 17,348, 13,434, 10,023, and 17,060)

New Zealand commercial deer farmers are paid based on the weight and quality of carcasses they produce, and an increase in lean muscle tissue production is needed to grow production efficiency. In order to investigate the possibility of capturing any beneficial variation in *MSTN* to improve muscle mass, variation in the intron 1 region of *MSTN* was investigated. This region was found to be variable in sheep and associated with variation in sheep meat carcass traits [7]; thus, it was investigated in 211 male New Zealand red deer, and its associations with selected phenotypic measurements were analyzed.

## 2. Materials and Methods

### 2.1. Animals Investigated and Phenotypic Data

Red deer (*Cervus elaphus*) from the Peel Forest Estate Stud (https://peelforestestate.co.nz accessed on 9 March 2022) in New Zealand were studied. The herd comprises over 8000 fully recorded, electronically ear-tagged deer, and was originally established with captured deer from Marshall Tito’s hunting reserve in Croatia and the Schulte-Wrede herd in Germany (which includes Romanian, Hungarian, and Czech bloodlines). The breeding system in the stud uses dedicated maternal sires to produce replacement breeding hinds and dedicated terminal sires to produce fast-growing progeny to maximize productivity. Both lines are bred for production using estimated breeding values produced by Deer Select (https://www.deernz.org/deer-hub/breeding/deer-select/, accessed on 9 March 2022), Wellington, New Zealand, and the maternal lines are now primarily based on English and German genetics.

Two hundred and eleven male deer from the maternal lines were investigated. These were born to hinds farmed in an extensive outdoor system on harder undeveloped hill country with a mixture of native grasses and aerial over sown grasses and clover, and were farmed together in paddocks on mixed ryegrass–clover pasture after weaning. All these animals were 2018 born male progeny from 21 sires. All the deer had their sire, date of birth, and a 12-month weight recorded. At approximately 11 months of age, corresponding to the spring molt, ultrasound scanning measurements were taken from a site over the *longissimus dorsi* (LD) muscle (eye muscle) between the 12th and 13th rib on the left side of the deer. Once restrained in a crush, each deer had a piece of fur over the scan site clipped to the skin and Aquasonic 100 gel (Parker Laboratories, Inc. Fairfield, NJ, USA) was applied to the scan site. The scanning was undertaken with a Medison SA600V ultrasound scanner and a 120 mm multi-frequency linear array probe operating at 3.5 MHz. The measurements recorded from the images were the maximum width and the maximum depth, the eye muscle image was manually traced using these points as a reference, and the cross-sectional area of the traced eye muscle (EMA) was calculated.

Because fawning hinds are left alone, as too much disturbance can lead to stress and abandonment, it would be challenging to record birth weights. We therefore presumed that the fawns were 10.2 kg at birth (an average reported for male red deer) [20], and this was used to calculate the average daily gain (ADG) from birth to 12 months of age.

### 2.2. Genomic DNA and Variation Screening

Genomic DNA samples in aqueous solutions for all the deer were obtained from AgResearch (Invermay, Mosgiel, New Zealand), with concentration ranging from 50.5 ng/µL to 155.8 ng/µL.

Variation in an *MSTN* intron 1 fragment was screened using a PCR-single strand conformation polymorphism (PCR-SSCP) technique. This region was selected as it was found to be polymorphic in sheep [7], because the Ensembl (www.ensembl.org, accessed on 9 March 2022) Rambouillet Sheep v1.0 genome construct (NM_001009428) describes 26 SNPs clustered at the 5′ end of this intron (no comparable analysis is available for red deer), and because in a human study [21], a nucleotide change in this region activates a cryptic splice site, leading to increased musculature. The DNA fragment was amplified using the PCR primers (5′-GAAACGGTCATTACCATGC-3′ and 5′-CATATTTCAGGCAACCAAATG-3′) that were designed for ovine *MSTN* [7], and the primers were synthesized using Integrated DNA Technologies (Coralville, IA, USA).

PCR amplification was performed in a 15-μL reaction containing approximately 50 ng of genomic DNA, 0.25 μM of each primer, 150 μM of each dNTP (Bioline, London, UK), 2.5 mM of Mg^2+^, 0.5 U of Taq DNA polymerase (Qiagen, Hilden, Germany), and 1× reaction buffer supplied with the enzyme. The amplification was undertaken in Bio-Rad S1000 thermal cyclers (Bio-Rad, Hercules, CA, USA), and the thermal cycling conditions included an initial denaturation for 2 min at 94 °C, followed by 35 cycles of 30 s at 94 °C, 30 s at 60 °C, and 30 s at 72 °C, with a final extension of 5 min at 72 °C.

The PCR amplicons were subject to SSCP analysis. Briefly, a 0.7-μL aliquot of each amplicon was mixed with 7 μL of loading dye (98% formamide, 10 mM EDTA, 0.025% bromophenol blue, 0.025% xylene-cyanol). After denaturation at 95 ^o^C for 5 min, the samples were rapidly cooled on wet ice and then loaded on 16 cm × 18 cm, 14% acrylamide–bisacrylamide (37.5:1) (Bio-Rad) gels. Electrophoresis was performed using Protean II xi cells (Bio-Rad) in 0.5× TBE buffer at 15 °C at 330 V for 19 h. The gels were silver-stained according to the method of Byun et al. [22].

### 2.3. DNA Sequencing and Sequence Analysis

Representative amplicons, which appeared to be homozygous based on the observed PCR-SSCP banding patterns, were purified using the MinElute PCR Purification Kit (Qiagen), and then sequenced in both directions at the Lincoln University DNA Sequencing Facility.

Sequence translations and comparisons were carried out using DNAMAN (version 5.2.10, Lynnon BioSoft, Vaudreuil, QC, Canada).

### 2.4. Statistical Analyses

Statistical analyses were performed using Minitab version 17 with a significance level of a = 0.05.

Univariate analyses were undertaken to ascertain the effect of sire and draft age on the eye muscle depth, the eye muscle width, the ewe muscle area, and the average daily gain. Next, general linear models (GLMs) were used to assess the effect of *MSTN* genotype on the eye muscle measurements and average daily gain. Sire was fitted as a random factor in the models, and draft age was fitted to the models for the eye muscle traits. The models used multiple pair-wise comparisons of the three genotypes (*AA*, *AB* and *BB*) with Bonferroni corrections to accommodate repetitive testing.

## 3. Results

**Figure 1:**
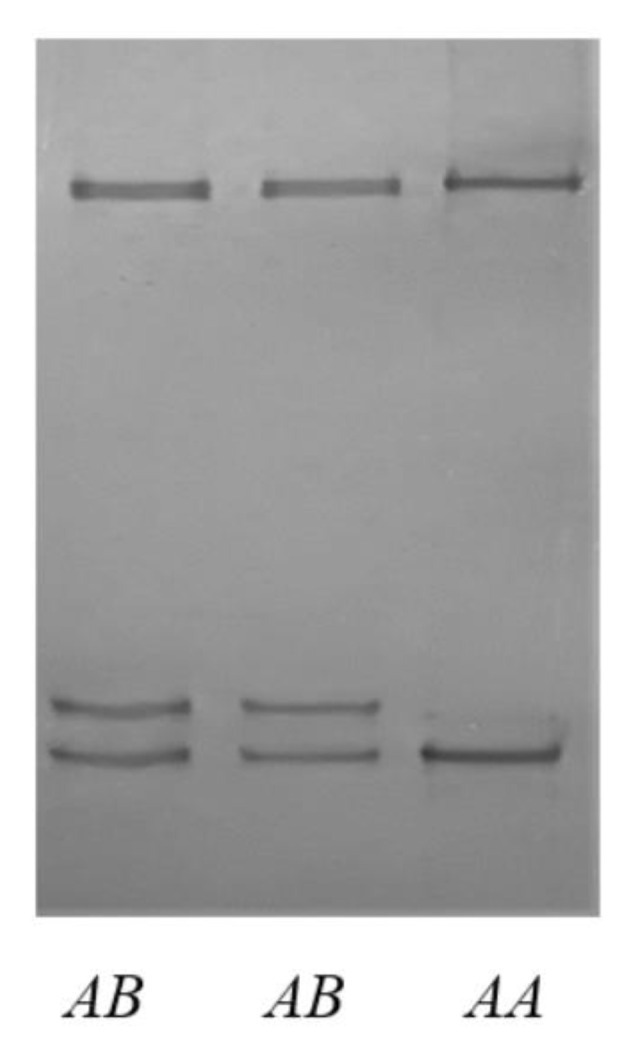
PCR-SSCP banding patterns for deer *MSTN* intron 1. Two banding patterns representing two variants *A* and *B* are shown in either homozygous or heterozygous forms.

**Figure 2:**
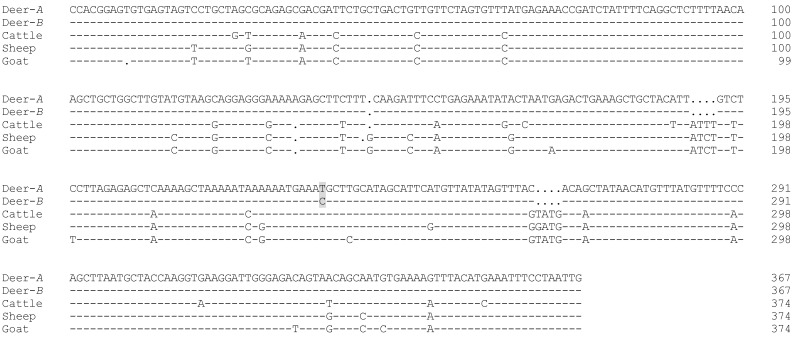
Alignment of two *MSTN* intron 1 variant sequences from red deer, together with the representative sequences reported in cattle, sheep, and goat. The variant sequences represent two unique PCR-SSCP patterns shown in Figure 1 and labelled as Deer-*A* and Deer-*B*, with the nucleotide difference between these two variants being shaded. The sequences exclude the PCR primer-binding sequences. The cattle, sheep, and goat *MSTN* sequences were retrieved from GenBank with accession numbers AB076403, DQ530260, and EF591039, respectively. Dashes represent nucleotides identical to the top sequence and dots are introduced to improve the alignment.

Two unique SSCP banding patterns were observed for the PCR amplicons of *MSTN* intron 1 in the New Zealand red deer investigated (Figure 1). Sequencing of representative PCR amplicons revealed two different DNA sequences (labelled as Deer-*A* and Deer-*B*; Figure 2). The sequence Deer-*A* was identical to the red deer genome assembly OU343110, while the sequence of Deer-*B* had one nucleotide sequence difference at c.373+224.

All three possible genotypes were found in the red deer, with *AA* being the most common genotype (at a frequency of 63.5%), followed by *AB* (at a frequency of 30.8%), and the least common genotype being *BB* (at a frequency of 5.7%). This gave frequencies of 78.9% and 21.1% for variants *A* and *B*, respectively.

With the 211 male red deer investigated, there was no difference between genotypes for eye muscle depth (*p* = 0.961), eye muscle width (*p* = 0.932), eye muscle area (*p* = 0.857), and average growth rate to 12 month of age (*p* = 0.912) (Table 1), which suggested there was no effect of the *MSTN* variation on the traits.

## 4. Discussion

This study reports sequence variation in red deer *MSTN*, but the level of variation detected is low, with only one single nucleotide polymorphism (SNP) being found in the intron 1 region that was typed and sequenced. In comparison, within this same region in sheep, nine SNPs are detected [6,7], and the Ensembl variation database records over 20 SNPs in cattle and four SNPs in goats.

The low level of sequence variation detected in these New Zealand red deer may be for several reasons. Firstly, while derived from multiple sires in a very large breeding herd, which has its origins in deer derived from Europe and the UK, the population studied may not be truly representative of the wider New Zealand or global red deer population. These deer might still in some way be related despite being the progeny of 21 sires, albeit the observation that one of the two sequences obtained for the amplified region precisely matched the reference genome sequence suggests that there is very little sequence variation in this region of *MSTN*. In this respect, the origin of the genome sequence OU343110 was a female red deer from the Reediehill Deer Farm in Scotland, which had DNA collected by researchers from the University of Edinburgh in February 2020 and with the genome assembly undertaken by the Wellcome Sanger Institute (Saffron Walden, United Kingdom). Reediehill Farm has provided the foundation stock for deer farming enterprises in the UK, Europe, USA, the Far East, and New Zealand, albeit a link between Reediehill and the deer in this study could not be found. It is therefore possible that greater variation may be found if more deer from across the globe are investigated.

It is also possible that the New Zealand red deer population could be a narrow gene pool to begin with, and that the importation of new genetics is uncommon. This seems unlikely as there is ongoing importation of genetics from England, Germany, and the rest of Europe into the breeding herd at Peel Forest Estate. Although there may have been a founder effect historically as only a select few animals were bought into New Zealand as the foundation of the wild population [23], more deer were subsequently bought into New Zealand during the establishment of deer farming. Semen importation and artificial insemination are now being used to introduce new genetics [24], so the likelihood of a founder effect resulting in reduced genetic diversity seems unlikely.

The investigated intron 1 region, whilst variable in other ruminant species, may not be so variable in red deer. This is supported by a DNA sequence comparison of this gene region from red deer, cattle, sheep, and goat (Figure 2). The sequence of this intron 1 region is very similar across cattle, sheep, and goat, but notable differences are observed in red deer, including nucleotide differences and insertions/deletions. It is possible that this gene region may be under different structural or functional constraints in different species; hence, different degrees of variation are allowed and thus detected. Future studies on other regions of the *MSTN* gene from red deer and other deer species may shed light on this.

Finally, natural selection might have acted on red deer *MSTN*. Myostatin not only affects muscling, but also other important phenotypes including locomotion and reproductive performance [25], and it has been observed that the tendons in *MSTN*-knockout mice are small, brittle and hypocellular [26]. Deer are powerful and agile animals, and anything that reduced the strength and flexibility of tendons could result in increased injury rates and death. Accordingly, over the course of evolution in the wild and subsequently in farming, any variation in *MSTN* that reduced fitness may have been selected against. Additionally in cattle, many of the mutations in *MSTN* result in muscle hypertrophy, but muscle hypertrophy is reported to associated with increased incidence of dystocia, so that assistance in delivery is often needed [27]. In a wild environment with deer, this would likely kill both the dam and fawn, and accordingly would be selected. There have also been links demonstrated between mutated alleles of *MSTN* and reduced fertility [28], and accordingly mutated animals may not be able to pass down their genetics to the next generation. Together, this suggests that variation in red deer *MSTN* might have been selected against in wild populations, and that only those that do not affect the function or potency of myostatin might have been maintained, given that deer were only domesticated about 50 years ago [19]. The finding of only one SNP in this intron region in red deer appears to support this contention.

Given the low level of sequence variation revealed in intron 1 of *MSTN* in the deer studied here, we recommend that more red deer are studied from herds across the globe, and that other coding and non-coding regions of *MSTN* are sequenced in those deer in the quest to find genetic variation.

## Figures and Tables

**Table 1:** Phenotypic data of New Zealand red deer between genotypes of the MSTN intron 1 region.

Trait	Raw Data (Mean ± SD)	Predicted Mean ± SE			*p* Value ^#^
		*AA* (*n* = 134)	*AB* (*n* = 65)	BB (*n* = 12)	
Eye muscle width (mm)	109.3 ± 5.23	108.9 ± 0.63	109.0 ± 0.75	109.4 ± 1.58	0.961
Eye muscle depth (mm)	41.7 ± 3.20	41.5 ± 0.28	41.7 ± 0.38	41.6 ± 0.83	0.932
Eye muscle area (cm^2^)	32.2 ± 3.20	32.2 ± 0.33	32.0 ± 0.42	32.4 ± 0.91	0.857
Average daily gain (g/d)	335.5 ± 23.7	335.0 ± 3.15	333.6 ± 3.52	335.9 ± 7.26	0.912

^#^*p* < 0.05 derived from the GLMs with a Bonferroni correction fitted.

## Data Availability

The data presented in this study are available on request from the corresponding author.
